# Improving medical experts’ efficiency of misinformation detection: an exploratory study

**DOI:** 10.1007/s11280-022-01084-5

**Published:** 2022-08-12

**Authors:** Aleksandra Nabożny, Bartłomiej Balcerzak, Mikołaj Morzy, Adam Wierzbicki, Pavel Savov, Kamil Warpechowski

**Affiliations:** 1 Gdańsk University of Technology, Gdańsk, Poland; 2 Polish-Japanese Academy of Information Technology, Warsaw, Poland; 3 Poznań University of Technology, Poznań, Poland

**Keywords:** e-health, Misinformation, Text-mining, Human-in-the-loop, Credibility assessment, Natural language processing, Machine learning

## Abstract

Fighting medical disinformation in the era of the pandemic is an increasingly important problem. Today, automatic systems for assessing the credibility of medical information do not offer sufficient precision, so human supervision and the involvement of medical expert annotators are required. Our work aims to optimize the utilization of medical experts’ time. We also equip them with tools for semi-automatic initial verification of the credibility of the annotated content. We introduce a general framework for filtering medical statements that do not require manual evaluation by medical experts, thus focusing annotation efforts on non-credible medical statements. Our framework is based on the construction of filtering classifiers adapted to narrow thematic categories. This allows medical experts to fact-check and identify over two times more non-credible medical statements in a given time interval without applying any changes to the annotation flow. We verify our results across a broad spectrum of medical topic areas. We perform quantitative, as well as exploratory analysis on our output data. We also point out how those filtering classifiers can be modified to provide experts with different types of feedback without any loss of performance.

## Introduction

The spread of medical misinformation on the World Wide Web is a critical problem in today’s society. We face a global ”infodemic” of outright health-related falsehoods, conspiracy theories, and dubious medical advice circulating in social media. The recent SARS-CoV-2 pandemic has exacerbated the existing distrust in pharmaceutical companies, low confidence in medical science, medical institutions, and governmental agencies responsible for public health [19, 32]. On the other hand, more and more people rely on online health information for self-treatment [6] while lacking the necessary skill to evaluate the credibility of such information. Given the possible consequences of using online health advice ungrounded in medical science, the task of aiding Web users in assessing the credibility of online health information becomes a high priority.

Distinguishing between credible and non-credible online medical information poses a substantial challenge even for experienced medical professionals, and even more so for ordinary Web users whose evaluation may be impacted by cognitive biases or psychological factors [1, 34]. Labeling source websites as either credible or non-credible is insufficient since false claims can be a part of an article originating from a credible source and vice versa. Often, disinformation is woven into factually correct medical statements that serve as camouflage. Even subtle changes to the wording, strength, or overtone of a medical statement can change its meaning, for instance, by exaggerating the side effects of a drug or by conflating relative and absolute risks of a medical procedure. As an example, consider the following phrase: *”Aspirin should not be consumed during pregnancy”*. This phrase is generally true but does not apply to an early pregnancy at risk of miscarriage — then, consuming small doses of aspirin can significantly lower the risk. The credibility of medical statements may also significantly depend on the context. For example, the phrase *”For starters, statin drugs deplete your body of coenzyme Q10 (CoQ10), which is beneficial to heart health and muscle function”*, despite factual correctness, would raise objections from medical professionals as it may discourage a patient from taking statins. In this example, the expert uses external knowledge from their clinical practice that benefits provided by statins far outweigh the potential risks associated with coenzyme Q10 deficiency for patients requiring statin therapy. This additional context of online health information evaluation makes it extremely difficult to frame the task in terms of machine learning.

Because assessing the truthfulness of medical statements is subjective, context-dependent, and challenging, in our research we formulate a different task for machine learning models: that of credibility evaluation. *Credibility* is a concept that can depend on the truthfulness of information, but also on other aspects, such as the potential for causing harm or misleading persuasion [45]. Consequently, credibility also applies to statements that cannot be directly verified but may still be harmful or misleading.

We define a medical statement to be *non-credible* if the statement is not in accord with current medical knowledge or entices a patient to make harmful health-related decisions, or inspires actions contrary to the current medical guidelines. We also use the general term *misinformation* to represent information that is not credible (regardless of the intention of the author, which may be malicious or benign).

Because of the critical costs of errors, it is paramount that credibility evaluation of health-related Web content is performed or supervised by trained medical practitioners. Those can be annotators who curate training data for statistical models or experts who provide final scores. Unfortunately, such experts’ availability, time and attention are scarce resources. Over-worked medical practitioners struggle to secure the time required for debunking online medical falsehoods and cannot keep up with the flood of online medical misinformation. Scarce human resources, stifling automatic online assessment methods, are the bottleneck. To address this issue, we propose to frame the problem of online health information evaluation as a machine learning problem. We formulate the business objective as the optimization of the utilization of medical experts’ time.

Such business objective has yet to be formulated as an objective function driving the training of statistical models. We treat the total time budget of a medical expert for debunking online medical information as a fixed value. Similarly, we treat the average time required by a medical expert to evaluate a single medical statement as a fixed value (the results of our experiments indicate that the average time to evaluate a statement by an expert is about 30 seconds). On average, a medical expert will evaluate a fixed number of statements. Optimizing the expert’s time utilization means increasing the proportion of non-credible statements discovered within her/his time budget.

We propose to focus medical experts’ attention on statements that are presumably non-credible and contain medical misinformation. This, in turn, requires the development of methods for the automatic discovery of credible statements. The objective is to maximize the precision with respect to non-credible medical statements (precision for the negative class) at a fixed, high precision threshold of filtering credible statements (precision for the positive class). In this way, we can extract a large set of medical statements which are guaranteed to contain credible medical information due to fixed precision and remove these statements from the queue of statements for human annotation, allowing medical experts to focus their limited time on the discovery of non-credible statements. Our experiments show that this approach increases the utilization of medical experts’ time by the factor of 2.

Our main contributions presented in this paper include:

- introduction of a general framework to optimize the utilization of medical experts’ time when annotating data for downstream training of machine learning models,
- evaluation of the framework on the task of medical misinformation annotation,
- developing a set of filtering classifiers for assessing the credibility of medical statements with the precision ranging from 83.5% to 98.6% for credible statements across ten different medical topics,
- analysis of most significant features that are used by filtering classifiers,
- providing human-interpretable explanations of filtering classifiers.

## Related work

There are multiple strategies for improving the credibility of online health information. They include information corrections, both automatically-generated and user-generated [4], and the manipulation of the visual appeal and presentation of medical information [11]. A recent meta-analysis [41] shows, however, that the average effect of correction of online health information on social media is of weak to moderate magnitude. The authors point out that interventions are more effective in cases when misinformation distributed by news organizations is debunked by medical experts. When misinformation is circulated on social media by peers, or when non-experts provide corrections, interventions have low impact.

The approaches to automatic classification of online medical misinformation differ depending on the media and content type. Most studies employ content analysis, social network analysis, or experiments, drawing from disciplinary paradigms [42]. Online medical misinformation can be effectively classified by using so-called peripheral-level features [48] which include linguistic features (length of a post, presence of a picture, inclusion of an URL, content similarity with the main discussion thread), sentiment features (both corpus-based and language model-based), and behavioral features (discussion initiation, interaction engagement, influential scope). Peripheral-level features proved to be useful for detecting the spread of false medical information during the Zika virus epidemic [10, 38]. Stylistic features can be used to identify hoaxes presented as genuine news articles and promoted on social media [33]. Along with identifying hoaxes, it is possible to identify social media users who are prone to disseminating these hoaxes among peers [13]. An applied machine learning-based approach, called *MedFact*, is proposed in [37], where the authors present an algorithm for trusted medical information recommendation. The *MedFact* algorithm relies on keyword extraction techniques to assess the factual accuracy of statements posted in online health-related forums.

More advanced methods of online medical information evaluation include video analysis (extracting medical knowledge from YouTube videos [22]), detecting misinformation based on multi-modal features (both text and graphics [43]), and website topic classification. The latter approach was successfully applied by [2, 21] using topic analysis (either Latent Dirichlet Annotation or Term-Frequency). Alternatively, text summarization may be used for this purpose [3]. In addition, Afsana et al. use linguistic features, such as word counts, named entities, semantic coherence of articles, the Linguistic Inquiry Word Count (LIWC), and external metrics such as citation counts and Web ranking of a document. A similar multi-modal approach is presented by Dhoju et al. [9] to distinguish with very high precision between reliable and unreliable media outlets publishing health-related information. Also, Wagle et al. use multi-modal analysis to evaluate the credibility of health & beauty blogs by analyzing the credibility of the platform, author, and images embedded in the blog [40].

An important aspect of our approach is the interpretability and explainability of filtering classifiers [27]. The description of recent advances in the field of machine learning interpretability is beyond the scope of this paper, interested reader is referred to a very thorough survey of explainable methods for supervised learning [5] and to an excellent book by Molnar [25]. In our work we utilize the Local Interpretable Model-agnostic Explanations (LIME) [35] technique to gain insights into features used by filtering classifiers to identify credible statements. LIME is an example of the black-box approach to model interpretability. Other popular black-box approaches include using Shapley values [24], partial dependence plots [12], and Morris sensitivity analysis [16, 26]. Alternatively, glass-box models can be used to explain algorithmic decisions of machine learning models. The most popular approaches include decision tree-based explainers [15], using Boolean rules to identify target classes [7], and Explainable Boosting Machines [23]. Implementations of many rule-based glass-box models are readily available in the imodels library [39].

This paper is the extension of work originally presented during the 22th International Conference on Web Information Systems Engineering WISE’2021 [28]. The original paper focused on improving the utilization of human annotators’ time when manually annotating the credibility of medical statements. This work extends previous report in a number of dimensions. We broaden the related literature review, in particular discussing relevant work on explainable machine learning models. We make a detailed report on annotation times recorded during the experiments. We add transformer-based models to the evaluation (BioBERT) and we include the results of these models in the summary of experiments. We present a new section pertaining to the generalization capabilities of tested models. The entire new section is devoted to the issue of explainability of models: we apply LIME to our filtering classifiers and we compare these explanations with more traditional approach based on Logistic Regression coefficient analysis. Detailed reports on the experimental results (TPOT configurations, Logistic Regression per topic) are included in two appendices.

## Methods

In this section we introduce the dataset compiled as the result of our project. We describe the annotation protocol and the annotation procedure, albeit in an abridged manner. For the detailed description of the dataset and the annotation process we refer the reader to [29]. We also present the augmentations applied to the data and the set of features used to train filtering classifiers. We conclude the section with the short overview of the training procedure and the introduction of explainable models used in the experiments.

### Dataset

We consider the credibility prediction of the full article as an insufficiently defined task burdened with source bias. That is why, instead of articles, we chose to classify smaller chunks of text (triplets of sentences, in particular). In previous approaches, the classifiers rated entire documents. For example, in the study evaluating entire articles [2], they were assessed against 10 criteria, none of which directly determines whether the content is credible or not. Our method differs from the approaches presented in the literature earlier in two important aspects: we leverage the context of medical expert’s annotation by data and label augmentation, and we modify the objective function to optimize for the recall of the positive class given the fixed precision threshold.

Our dataset consists of over 10000 sentences extracted from 247 online medical articles. The articles have been manually collected from health-related websites. The choice of major categories (cardiology, gynecology, psychiatry, and pediatrics) has been dictated by the availability of medical experts participating in the experiment. After consulting with medical experts, we have selected certain topics known to produce controversy in online social networks. For each topic, we have collected a diversified sample of articles presenting contradicting views (either supportive or contrarian) and we have extracted statements for manual evaluation by medical experts. The dataset is open-sourced and publicly available.^1^

Nine medical experts took part in the experiment, including 2 cardiologists, 1 gynecologist, 3 psychiatrists, and 3 pediatricians. All experts have completed 6-years medical studies and then a 5-year residency program. The experts were paid for a full day of work (approximately 8 hours each). Each medical expert had at least 10 years of clinical experience, except for the gynecologist who was a resident doctor. We have accepted his participation in the experiment due to his status as a Ph.D. candidate in the field of medicine. One of the psychiatrists held a Ph.D. in medical sciences. Given the high qualifications of participants, we consider their judgments as the ground truth for medical statement evaluation. The experts were allowed to browse certified medical information databases throughout the experiment. Each expert evaluated the credibility of medical statements only within their specialization.

**Table 1:** Number of sentences from each class by the topic

Category	Topic	CRED	NEU	NONCRED
Cardiology	Antioxidants	375	175	144
Cardiology	Heart supplements	221	124	78
Cardiology	Cholesterol and statins	1058	565	406
Gynecology	Cesarean section vs. natural birth	275	53	31
Pediatry	Children & antibiotics	298	52	82
Pediatry	Diet and Autism	236	71	124
Pediatry	Steroids for kids	560	101	40
Pediatry	Vaccination	730	223	309
Pediatry	Allergy testing	790	398	214
Psychiatry	Psychiatry	1194	676	402

Collected online articles were automatically divided into sentences and presented to the medical experts in random order. Sentence segmentation has been done using the dependency parser from the spaCy text processing library. Since input text follows closely the general-purpose news style, the default spaCy processing pipeline produces very robust sentence segmentation. Along with each sentence we have displayed a limited number of automatically extracted keywords. If the medical expert decided that a sentence could not have been assessed due to insufficient context, he or she could have expanded the annotation view by showing preceding and succeeding sentences. Each medical expert was asked to annotate approximately 1000 sentences. Medical experts evaluated the credibility of sentences with the following set of labels and the corresponding instructions:

**Table 2:** Number *m* of surrounding sentences needed to understand the context and evaluate the credibility of a sentence for credible, non-credible, neutral, and all sentences

m	Credible [%]	Non-credible [%]	Neutral [%]	All [%]
0	80.07	71.27	88.30	**80.43**
1	18.83	26.60	11.03	**18.39**
> 1	0.18	0.37	0.04	**0.18**

- CRED (credible) — a sentence is reliable, does not raise major objections, contains verifiable information from the medical domain.
- NONCRED (non-credible) — a sentence contains false or unverifiable information, contains persuasion contrary to current medical recommendations, contains outdated information.
- NEU (neutral) — a sentence does not contain factual information (e.g., is a question) or is not related to medicine.

Table 1 presents the number of sentences in each class summarized by category and topic. Within the four larger topical categories (cardiology, gynecology, psychiatry, or pediatrics), our dataset is divided into smaller subsets (topics). Considering these topics separately dramatically improves the performance of the classifiers. However, some topics included in the dataset were too small for training a classifier. Thus, we do not consider them further in this article.

### Data augmentation

The annotation of the dataset by medical experts has revealed the importance of context for providing a label (see Table 2). Over 25% of non-credible sentences required the surrounding context of one sentence, with 20% of credible sentences and 12% neutral sentences requiring similar context. To provide this context for statistical models, we have decided to transform single sentences into sequences of consecutive non-overlapping triplets of sentences. Since individual sentences have already been labeled by medical experts, we have transferred ground truth sentence labels to triplet labels in the following way:

- negative: a triplet is negative if any of the sentences constituting the triplet has the label NONCRED,
- positive: a triplet is positive if all of the sentences constituting the triplet are either CRED or NEU.

Example of a positive triplet (from ”Statins & cholesterol”):

*”Not smoking could add nearly 10 years and quitting increases life expectancy by reducing the chances of emphysema, many cancers, and heart disease. Although my doctor checks my cholesterol every year, it remains low and taking a statin will have a very small, if any, effect on my life expectancy. What’s worse, my doctor has never asked if I smoke cigarettes, exercise regularly, or eat a healthy diet.”*

Example of a negative triplet (from ”Statins & cholesterol”):

*”OK, maybe the benefits of taking a statin are small, but many smart doctors say a reduction of five-tenths or six-tenths of 1% is worthwhile. Yet the few published observations on people over the age of 70 do not show any statistically significant statin-related reductions in deaths from any cause. Of course, not everyone is like me.”*

### Feature set

Features that have been selected for credibility classification purposes are based on the qualitative analysis of the dataset concerning the findings reported in Section 2. The ultimate number of features varies between categories. The feature set has been created manually and feature selection methods have been used to remove non-informative features. The choice of traditional NLP features has been deliberate as we want to maintain the explainability of filtering classifiers. However, we compare them to the compressed lexical features obtained by the state-of-the-art deep learning language model BioBERT [20] trained on clinical data.

#### Uncased TF-IDF (number of features: varying from 920 to 4103)

Bag of words, n-gram, term frequency (TF), term frequency inverted document frequency (TF-IDF) are the most commonly used textual features in natural language processing [47]. In this work, we chose TF-IDF values to account for the importance of each word. We use the Python package spaCy to perform sentence tokenization and lemmatization.

#### BioBERT vectors (number of features: up to 768)

BioBERT is a pre-trained language representation model for the medical domain. It was designed for linguistic tasks of Medical Entity Recognition, relation extraction, and question answering [8, 49]. The model we use was trained on a combination of general purpose and medical corpora (English Wikipedia, Books Corpus, PubMed Abstracts and PMC full articles). In our work, we decided to use the sentence vectorization module of BioBERT. This module transforms each paragraph in the corpus into a numerical vector. This vector is an aggregation of word embeddings generated for each word in the paragraph by the BioBERT model.

#### Dependency tree-labels count (number of features: up to 45)

Overly complex sentences have a higher probability to contain the hedging part than simple sentences (the base of a sentence may contain a factually false statement, but the other part would soften its overtone so that it seems credible). Thus, we count the base elements of dependency trees to model the potential existence of such phenomena.

#### Named entities counter (number of features: up to 18)

There are some indicators of conspiratorial and/or science-skeptical language (hence the popularity of using agent-action-target triples in the study of conspiratorial narratives [36]). Those narratives may be captured by counting named entities of specified categories, such as false authority (PERSON), Big Pharma blaming (ORGANIZATION, PRODUCT), distrust to renowned institutions (ORGANIZATION), facts and statistics (NUMBER). In the experiment we have used the NER labeling scheme available in the English language model offered by the spaCy library.

#### Polarity and subjectivity (number of features: 2)

Sentiment analysis is a broadly-used feature set for misinformation detection classifiers. It has been used, for example, for detecting anti- and pro-vaccine news headlines [46]. Highly polarized and/or emotional language can indicate misinformation Figs. 1, 2, 3, 4, 5, 6, 7, 8, 9, 10, 11, 12 and 13.

**Fig. 1:**
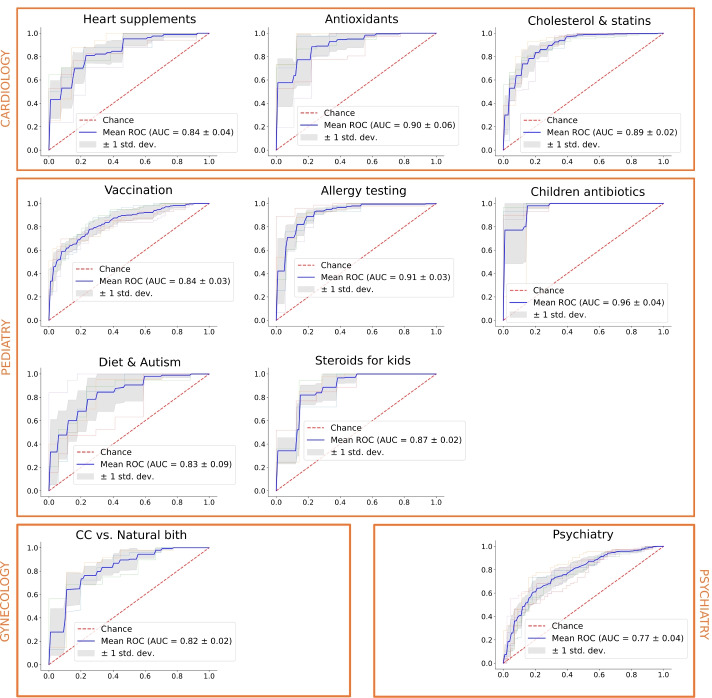
ROC curves of cross-validated classification results for each medical topic

#### LIWC (number of features: 93)

Aggressive, overly optimistic, advertising language (e.g. for a drug or novel therapy) or other patterns can affect the credibility of textual information [18]. The LIWC offers a corpus-based sentiment analysis approach by counting words in different emotion categories. Empirical results using LIWC demonstrate its ability to detect meaning in emotionality. In addition, it has been employed to extract the sentiment features for the detection of misinformation in online medical videos [17]. LIWC provides features regarding emotional dimensions, the formality of the language, spatial and temporal features, as well as structural information (e.g. word per sentence count).

### Feature selection and model training

The workflow for training statistical models is identical for each topic and includes two steps: feature selection and model selection. Feature selection is performed using Logistic Regression and Recursive Feature Elimination (RFE) [14]. RFE conducts a backward selection of features, starting from a predictive model using all available features. For each feature, the importance score is computed, and the least important feature is removed. The model is retrained with remaining features and the procedure is repeated until the desired number of features remains. We use Logistic Regression as the baseline model for RFE, limiting the number of features to 30% of the number of samples in a given topic. In this paper, we assume that the list of topics is known in advance and that each sentence is already assigned to a topic. This, of course, raises the question of the practical applicability of our method when the topic of an article is unknown. Recent advances in automatic medical subdomain classification [44] suggest that the topic of the article can be successfully extracted from the text.

We have also conducted model training on the unpruned feature set. The results were very disappointing, topical models performed on par with random classification. Thus, we do not include these models in the evaluation. The results for the unpruned feature set strengthen the intuition that credibility assessment is heavily domain-dependent. In our view, this has two consequences. Firstly, the prospects of training a universal credibility assessment model are unlikely as the credibility encoded in the syntax is limited. It seems that most of the credibility is hidden in semantically-loaded features that are specific to a topic. Secondly, the importance of subject matter experts in evaluating the credibility should not be ignored, because only these experts can properly evaluate the significance of topical features. It also stresses the need to augment credibility assessment models with explainability to assist the experts.

For training the model we use the TPOT library [31]. TPOT uses a genetic algorithm to optimize the workflow consisting of feature pre-processing, model selection, and parameter optimization, by evolving a population of workflows and implementing mutation and cross-over operators for workflows. To constrain the space of considered models we use Logistic Regression, XGBoost, and the Multi-layer Perceptron as the initial pool of available models. The optimization is driven by the *F*_1_ measure.

### Explainable models

#### Models generalization

We try to answer the question about the ability of the models to generalize between subdomains. To achieve that, we analyzed the most important features for all subdomains with an emphasis on the similarities between the domains (Table 5). We also calculated the percentage of stylometric features from the sets of the most important model features for each sub-domain (Table 4).

#### Feature weights from logistic regression

All pipelines selected by TPOT involve black-box classifiers and as such cannot be explained globally in terms of feature importance. Only local approximate explanations for individual samples may be generated by techniques such as SHapley Additive exPlanations (SHAP) [24] or Local Interpretable Model-agnostic Explanations (LIME) [35].

For those subdomains where the *F*_1_ measure and the *AUC* achieved by Logistic Regression were close to the performance of the pipeline chosen by TPOT (see Appendix A) we used the coefficients of the Logistic Regression models to estimate the importance of each feature and its contribution to the final predictions (see Section 4.4). This may be done since the features were scaled to unit variance.

#### Locally interpretable model-agnostic explanations

To gain better insight into how filtering classifiers work and boost medical experts’ confidence in the robustness of the filtering of credible statements, we perform additional analysis using the locally interpretable model-agnostic explanations (LIME) method [35]. LIME encapsulates any black-box model by a glass-box model (e.g. linear regression or decision tree) operating in the close vicinity of the currently explained instance. The features of the current instance are slightly perturbed (the perturbation type depends on the modality of the instance and may include masking a word or a part of an image, adding noise to the numerical value, flipping of a Boolean value, etc.). The glass-box model is trained only on a small set of perturbations, providing a local approximation of the global (and possibly black-box) model. As the result, the glass-box model identifies features of the explained instance that contribute the most to the current decision of the black-box model.

## Results

In this section we present the results of conducted experiments. We begin by discussing the process of manual data annotation and its limitations. We show how our active annotation approach optimizes the utilization of subject matter experts’ time by re-ranking annotation tasks. We briefly discuss the issue of model generalization, and we conclude the section with extensive analysis of the usefulness of model explainability in credibility assessment.

### Times needed to assess a single statement

During our experiment, we have measured the times required by experts to evaluate the credibility of medical statements. This information is of crucial importance in practice, as the average time to evaluate a statement can be used to determine the throughput of an expert. Of course, it is necessary to keep in mind that experts cannot work indefinitely, and need to take periodic breaks in order to rest.

**Fig. 2:**
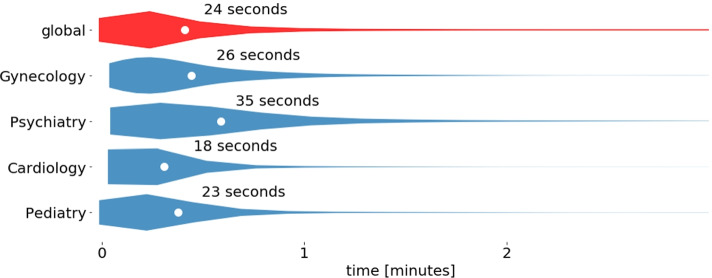
Times needed to assess a single statement by the medical expert. White dots indicate the average evaluation times, which are explicitly stated in seconds next to each distribution graph

Figure 2 shows the distributions of evaluation time for all statements, and for statements in the four main disciplines of our study: gynecology, psychiatry, cardiology, and pediatry. The distribution is long-tailed, but the longer times of statement evaluation are infrequent. Overall, the distributions differ for various topics from 18 to 35 seconds, depending on the topic (experts in cardiology are the fastest, while in psychiatry - the slowest). For an expert who works 8 hours per day, with periodic breaks of 15 minutes every hour (leaving 6 hours of effective working time), this gives an average number of evaluated statements per day in the range of 617 to 1200 statements. Recall that, on average, one article in our dataset has approximately 40 statements (there are 10000 statements from 247 articles). This means that an expert can evaluate from 15 to 30 articles per working day, depending on the topic of the article.

### Optimization of experts’ evaluation time

The main objective of our method is to maximize the utilization of medical experts’ time when annotating online medical statements. We optimize statistical models to find credible statements, thus increasing the number of non-credible statements that can be presented to medical experts. The results below analyze the efficiency of trained statistical models in finding credible statements. Recall from Section 3.2 that statistical models are trained on a binary dataset consisting of positive (credible and neutral) and negative (non-credible) triplets of sentences.

**Fig. 3:**
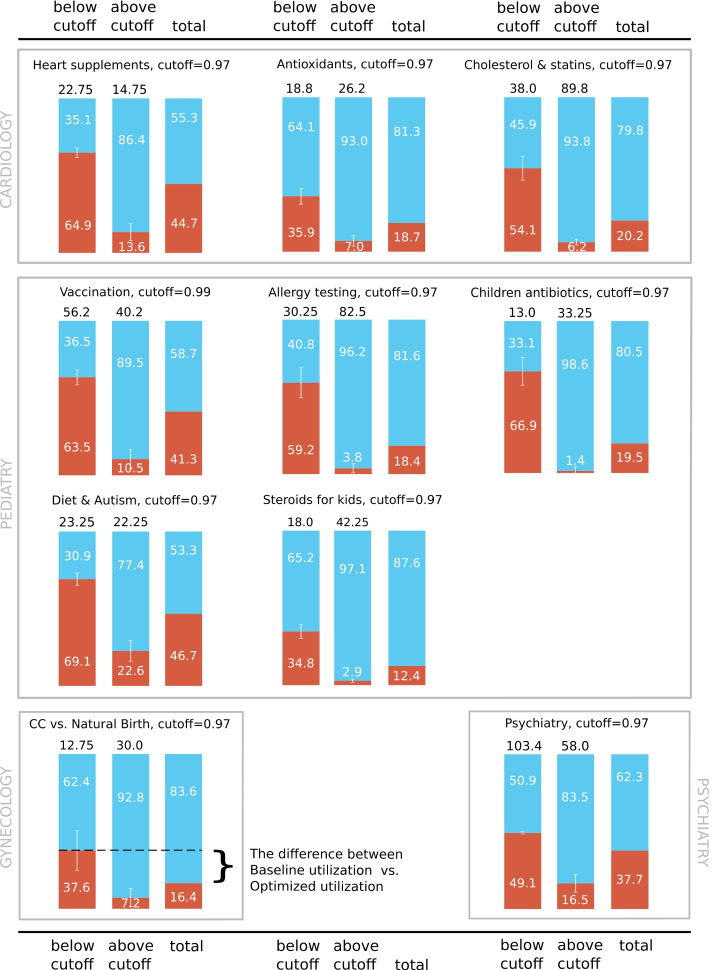
Cross-validated proportions of positive and negative samples (**a**) below the cutoff (**b**) above the cutoff (**c**) in the entire test set. This corresponds to precision for the negative class, precision for the positive class and total label proportions, respectively. Black labels indicate the mean number of samples in each group. Each bar has the standard deviation indicator (white vertical line)

Figure 1 presents ROC curves for cross-validation. The number of folds depends on the number of samples in a given topic. Based on the ROC curves we have empirically adjusted the cutoff threshold for each classifier’s prediction of the positive class. Our goal was to maximize the precision of the negative class while preserving fixed high precision for the positive class. In other words, samples that fall above the cutoff threshold are assumed to contain solely credible or neutral sentences, and will not be presented to medical experts for manual evaluation. We have selected the cutoff threshold for each topic using the following criteria:

- the difference between the proportion of true negative samples and the proportion of negative samples in the entire test set should be maximized, with minimum variance,
- the precision for the true positive class should be maximized,
- the number of samples above the cutoff should be maximized.

The results of the cutoff filtering are presented in Figure 3. For each topic, we show the distribution of positive and negative samples in the entire topic (the *total* column) and in the subsets defined by the cutoff. This corresponds to precision for the negative class (left bar), precision for the positive class (middle bar), and total label proportions (right bar). For instance, there are 44.7*%* of negative samples and 55.3*%* of positive samples in the *Heart supplements* topic. The subset of samples defined by the cutoff point of 0.97 contains only 13.6*%* of negative samples, and the remaining subset contains 64.9*%* of negative samples. In other words, by removing the samples above the cutoff threshold from manual experts’ evaluation we are increasing the number of negative samples that the experts may annotate from 44.7*%* to 64.9*%*. We refer to the proportion of negative samples in the topic as the *baseline utilization*, and the proportion of negative samples after the intervention (i.e., below the cutoff threshold) as the *optimized utilization*.

In Table 3 we report baseline utilization, the difference in percentage points with respect to the optimized utilization, and the factor of improvement of medical experts’ time utilization. Those values are reported for both models: with TF-IDF and BioBERT lexical features. We denote the percentage point difference value as the *pp. improv.* - percentage point improvement, as for each topic the difference is in favor of using our filtering classifiers.

**Table 3:** Comparison of baseline and optimized utilization of medical experts’ time

Category	Baseline utilization [%]	pp. improv. [TF-IDF]	factor [TF-IDF]	pp. improv. [BioBERT]	factor [BioBERT]
A	44.7	20.2	1.5	**27.6**	**1.6**
B	18.7	17.2	1.9	**30.7**	**2.6**
C	20.2	**33.9**	**2.7**	21.9	2.1
D	41.3	22.2	1.5	**28.1**	**1.7**
E	18.4	**40.8**	**3.2**	17.0	1.9
F	19.5	**47.4**	**3.4**	35.7	2.8
G	46.7	**22.4**	**1.5**	12.2	1.3
H	12.4	22.4	2.8	**26.8**	**3.2**
I	16.4	21.2	2.3	**25.0**	**2.5**
J	37.7	11.4	1.3	12.9	1.3
Mean	–	**25.9**	**2.2**	23.8	2.1

Results presented for both models: (1) using TF-IDF and (2) BioBERT vectors as lexical features. A - heart supplements; B - Antioxidants; C - Cholesterol & statins; D - Vaccination; E - Allergy testing; F - Children antibiotics; G - Diet & Autism; H - Steroids for kids; I - C-section vs. Natural Birth; J - Psychiatry

### Models generalization

Table 4 presents the distribution of significant features between feature sets for TF-IDF and BioBERT-based models. Generally speaking, models built upon TF-IDF vectors are topic-specific, which may indicate the need for manual fact-checking. However, there are subdomains where the participation of the stylometric features is significant, e.g. *’antioxidants’*. It may be the result of the specificity of this category, where many of the texts were advertisements of either valid or dubious substances.

A much greater share in building filtering classifiers (up to 50% in the case of the category *’heart supplements’*) is when we apply stylometric features along with compressed lexical features, i.e., when the text is embedded using representations extracted from a language model such as BioBERT. Although we lose the ability to directly interpret model decisions related to lexical features (it is not possible to explicitly interpret BioBERT vector’s dimension values), we gain a much greater share of meaningful stylometric features in model construction. There seems to exist a trade-off between lexical and stylometric model explainability, we either explain an algorithmic decision based on lexical features, or based on stylometric features, but not both.

Particularly noteworthy are those stylometric features which have a large share in building filtering classifiers based on BioBERT representations, in particular in the case of categories where models based on BioBERT outperformed models based on TF-IDF. Those models include (per category): statins, antioxidants, vaccination, steroids for kids, C-section vs. natural birth, and (although insignificantly) psychiatry. The features particularly involved in model creation include mostly LIWC features, but also tags retrieved from dependency parsing.

From Table 5 we can see that there are not many stylometric features that are common to all categories (for models built upon TF-IDF vectors). This may indicate that models should be prepared for coherent datasets of very narrow domains.

**Table 4:** Percentage of stylometric features from the sets of the most important model features for each sub-domain

Category	LIWC	NER	POS	DEP	Sent	Lexical
TF-IDF						
statins	5.3%	0.0%	0.5%	0.5%	0.5%	93.2%
vaccines	2.8%	0.7%	0.7%	1.4%	0.0%	94.4%
psychiatry	4.3%	0.0%	0.0%	0.0%	0.0%	95.7%
allergy testing	8.2%	0.0%	0.0%	0.0%	0.0%	91.9%
antioxidants	14.7%	0.0%	0.0%	0.0%	0.0%	85.3%
steroids for kids	12.3%	0.0%	0.0%	0.0%	0.0%	87.7%
children antibiotics	3.1%	0.0%	0.0%	0.0%	0.0%	96.9%
diet and autism	5.5%	0.0%	0.0%	0.0%	0.0%	94.5%
heart supplements	12.0%	2.0%	0.0%	0.0%	0.0%	86.0%
cc vs. nb	3.9%	0.0%	0.0%	0.0%	0.0%	96.1%
BioBERT						
statins	12.1%	3.2%	3.2%	4.2%	0.5%	76.8%
vaccines	13.9%	2.9%	2.9%	10.0%	0.7%	69.7%
psychiatry	10.7%	0.0%	2.9%	3.6%	0.0%	82.9%
allergy testing	10.4%	3.00%	2.2%	4.4%	0.0%	80.0%
antioxidants	14.7%	1.4%	0.0%	0.0%	0.0%	84.0%
steroids for kids	21.5%	0.0%	2.8%	8.3%	0.0%	67.4%
children antibiotics	13.9%	3.1%	3.1%	10.8%	0.0%	69.2%
diet and autism	14.6%	1.8%	1.8%	5.5%	0.0%	76.36%
heart supplements	22.0%	8.0%	2.0%	16.0%	2.0%	50.0%
cc vs. nb	22.0%	0.0%	2.0%	14.0%	4.0%	58.0%

LIWC - Linguistic Inquiry Word Count; NER - Named entities count; POS - parts of speech count; DEP - dependency parsing elements count; sent - either polarity or subjectivity of the text; lexical - features that are not stylometric, retrieved either by TF-IDF transformation or the BioBERT model

**Table 5:** Number of appearances of those stylometric features that appear more than once per category

Feature name	Number of appearances
Long words (more than 6 letters)	4
Certainty (words such as ”always”, ”never”)	3
Emotional tone	2
First person plural count	2
First person singular count	2
Adjectives count	2
Causation (words such as ”because”, ”effect”)	2
Past focus (words such as ”ago”, ”did”, ”talked”)	2
Health-related words (”clinic”, ”flu”, ”pill”)	2
Assent words (”agree”, ”OK”, ”yes”)	2
Period count	2
Cognitive processes indicators (words such as ”cause”, ”know”, ”ought”)	2
Ingestive processes indicators (words such as ”dish”, ”eat”, ”pizza”)	2

### Explainable models

For all sub-domains in Appendix A, we present models selected by TPOT. We compare the results of the winning models with the base model, the logistic regression. There are often cases where the logistic regression obtained only slightly worse results than the selected models. For such cases, we assumed that the weights of the logistic regression features are suitable for general explanations of the filtering classifiers’ decisions. Feature weight charts for logistic regression for all of the above-defined cases are shown in the Appendix B. Here we present exemplary explanations of models for two topics, antibiotics and dieting in autism, to illustrate the usefulness of having human-interpretable explanations of algorithmic decisions.

#### Children antibiotics

**Fig. 4:**
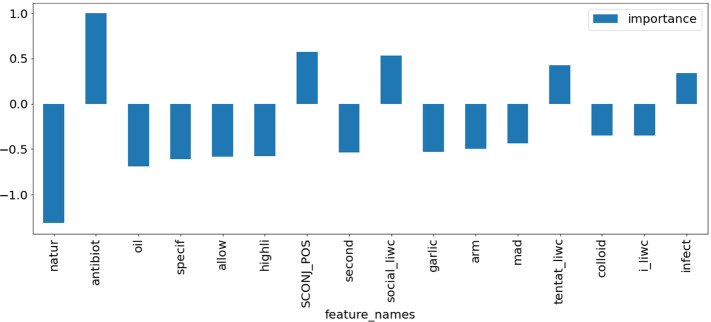
Feature weights retrieved from Logistic Regression model for ’children antibiotics’ category. Top absolute 16 feature weights are depicted

Figure 4 presents the most important features for distinguishing between credible and non-credible statements regarding the use of antibiotics in children. Features that contribute to the credibility of statements include the use of the word *antibiotic*, the presence of subordinating conjunctions (which characterize complex sentences with constituent subordinate clauses), the presence of ”social” vocabulary (i.e., words related to family and friends), as well as the presence of words marking tentative statements (*maybe, perhaps*). On the other side, non-credible statements are characterized mostly by the presence of specific keywords (*nature, oil, allow, garlic, colloidal silver*). Interestingly, the only keyword marking credible statements is *infection*, which is probably the term avoided by people opposed to the use of antibiotics in children.

Consider the following statement: *”However, this study did not determine whether antibiotic use is causally related to breast cancer or if other factors were involved. Certain antibiotics, such as methicillin, vancomycin, sulfonamides, gentamicin, fluoroquinolones, gatifloxacin, levofloxacin, moxifloxacin, and streptomycin, can be harmful for your kidneys. A 2013 study published in the Canadian Medical Association Journal found that there is an increase in risk of acute kidney injury among men with use of oral fluoroquinolones.”*

**Fig. 5:**
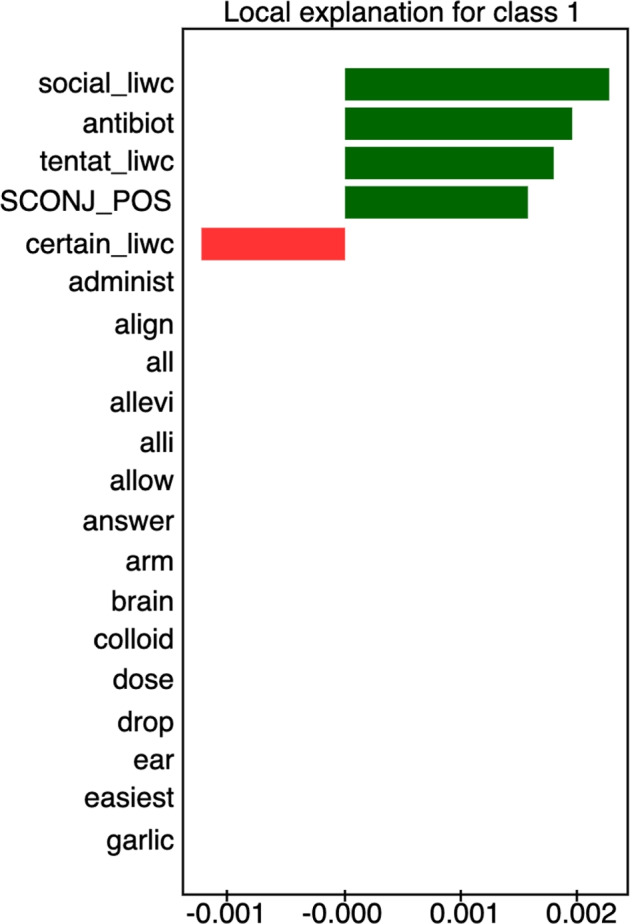
LIME explanation for a sentence on antibiotics

**Fig. 6:**
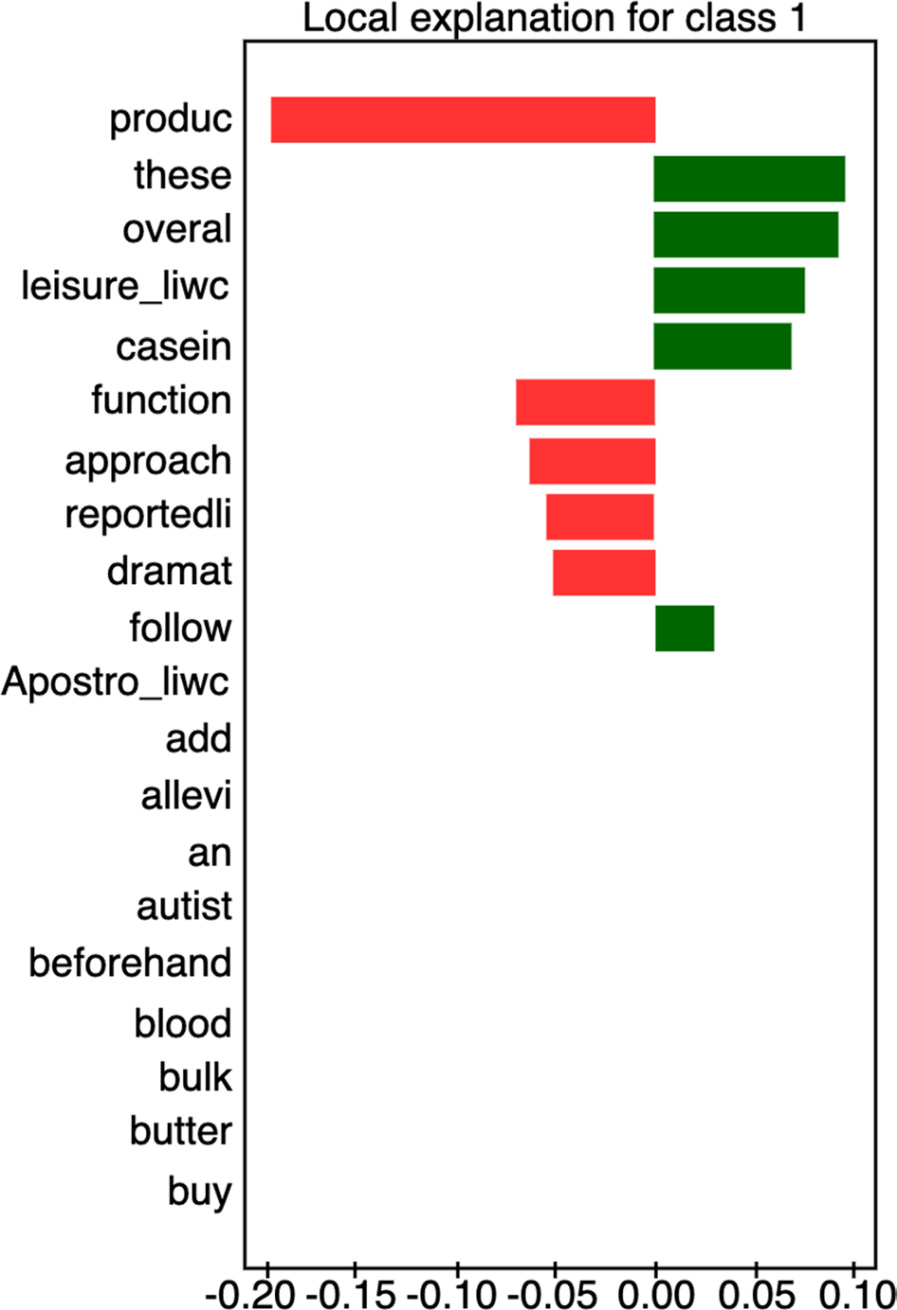
LIME explanation for a sentence on diet & autism

This sentence is credible and in line with the current medical knowledge. Figure 5 presents the explanation of the sentence generated by LIME. A medical expert can see that the main reason why this sentence has been classified as credible is the presence of the word *antibiotics* combined with complex phrase structure and tentativeness of the language (*however, whether, did not determine*).

#### Diet & autism

**Fig. 7:**
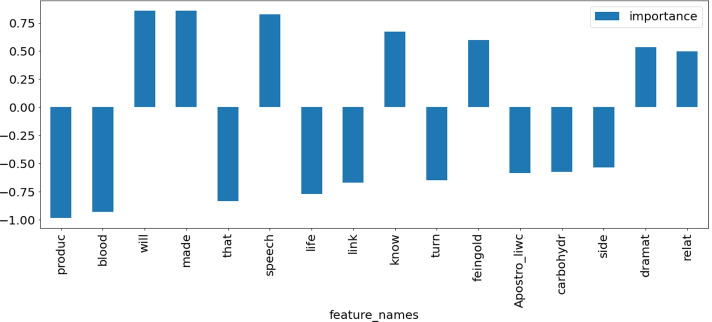
Feature weights retrieved from Logistic Regression model for ’diet & autism’ category. Top absolute 16 feature weights are depicted

Most discriminative features for classifying sentences as either credible or non-credible in the domain of diet and autism are depicted in Figure 7. One should remember that this particular subject is extremely sensitive as parents with autistic children may be more vulnerable to exploitation, or easier to accept scientifically unsound recommendations. Features characteristic of non-credible statements include very general terms (*product, blood, life, link, turn*) as well as, surprisingly, excessive use of apostrophes. Credible statements also share general terms (*will, made, know, speech, dramatic, relative*), but also mention the Feingold diet, a well-known elimination diet introduced by Benjamin Feingold in the 1970s.

Compare the example of a sentence on antibiotic use with the following non-credible sentence on diet & autism: *”These diets include the following: Casein-free diet (casein is a protein found in milk; this diet eliminates milk and all by-products of milk). In the case of the Autism Spectrum Disorders (ASDs), many parents have reported a reduction in autism symptoms when certain dietary interventions have been tried. For some children, dietary approaches have reportedly produced dramatic changes in overall functioning.”*

Figure 6 shows the LIME explanation of the sentence. The sentence is correctly classified as non-credible due to the presence of keywords (*product, function, approach, reported, dramatic*). Keywords associated with credibility (*these, overall, casein*) are not specific enough to sway the decision of the classifier.

## Discussion

Evaluation of the credibility of online medical information is a very challenging task due to the subjective assessment of credibility, and the specialized medical knowledge required to perform the evaluation [30]. Fully automatic classification of online medical information as credible or non-credible is not a viable solution due to the complex externalities involved in such classification. For the foreseeable future, keeping a human judge in the annotation loop is a necessity. At the same time, qualified human judges are the scarcest resource and their time must be utilized efficiently. Previous approaches to automatically assessing the credibility of medical texts did not take into account the need to weave a human judge into the real-time verification process.

In our work, we present a framework for the optimization of the utilization of medical experts’ time when evaluating the credibility of online medical information. To prioritize the evaluation of non-credible information by medical experts, we train classifiers that can filter out credible and neutral medical claims with very high precision exceeding 90*%* for most medical topics considered in our study (vaccination, allergy testing, children antibiotics, steroids for kids, antioxidants, cholesterol & statins, and C-section vs. natural birth).

Table 3 depicts the key benefit for the potential human-in-the-loop fact-checking system that our solution provides — an increase in the probability that a medical expert will encounter a non-credible medical statement in the annotation batch. As we can see, for all topics the improvement in the utilization of medical experts’ time is substantial. The average improvement over all topics is 25.9 percentage points, which means that within the same amount of time and at the same average time needed to annotate a single sentence, medical experts using our method annotate over two times as many non-credible medical statements on average. It is a ”pure win” since this improvement does not require any changes to either the annotation protocol or the annotation interface, we simply make much better use of the experts’ time allocated to data annotation.

In addition to the aforementioned important practical implications of using filtering classifiers to prioritize the evaluation of non-credible statements, these classifiers can explain their decisions in a human-interpretable way. Many practical conclusions can be drawn from general and local explanations. For example, the overwhelming share of topic-specific characteristics in classification may indicate that medical fake news are based on certain specific narratives (e.g., vaccines cause autism, high cholesterol is not an indicator of cardiovascular disease) that spread online by copying and pasting or copying and rewriting. This in turn may suggest focusing on semantic similarity measurements as a primary tool for medical fake news detection.

## Conclusions and future work

One limitation of our method is a certain number of statements that contain misinformation that would not be seen by experts. However, we need to keep in mind that medical experts may not see all statements anyway, as their limited time and attention are not enough to process all suspicious information.

In a realistic use-case scenario, medical experts would continually evaluate a stream of statements derived from the ever-growing set of online articles on medical and health topics, as well as information from social media. Our method increases the efficiency of misinformation detection by medical experts, who will discover more than twice as much misinformation without increasing the time spent on evaluation (or the number of evaluating experts), and without any changes to the annotation workflow. Our method can be regarded as a universal filter for medical Web content. Moreover, we show that we can modify the input features for the filtering classifiers to provide medical experts with different types of feedback, either lexical or stylometric, without any loss of performance. Because we cannot provide medical experts with both lexical and stylometric explanations, it remains to be examined which type of feedback is more useful for medical experts.

In our future work, we plan to focus on gathering more data by introducing the demo expert crowd-sourcing system in selected medical universities. We plan to emphasize the importance of the iterative process of adjusting proper annotation protocol and professional training for medical students. Our goal is to elevate medical students’ annotation accuracy to the expert level (like medical practitioners with at least a few years of experience), thus further reducing costs of expert medical credibility annotation.

## Figures and Tables

**Fig. 8:**
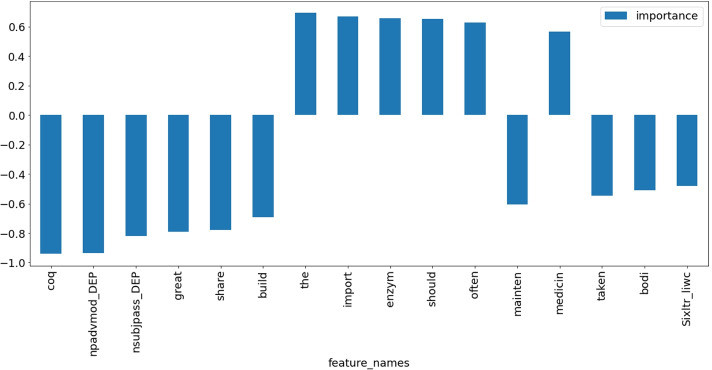
Feature weights retrieved from Logistic Regression model for ’heart supplements’ category. Top absolute 16 feature weights are depicted (roughly 30% of all model features)

**Fig. 9:**
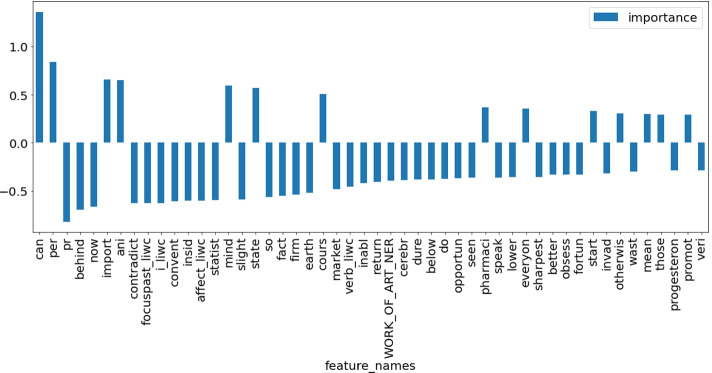
Feature weights retrieved from Logistic Regression model for ’statins’ category. Top absolute 40 feature weights are depicted (roughly 20% of all model features)

**Fig. 10:**
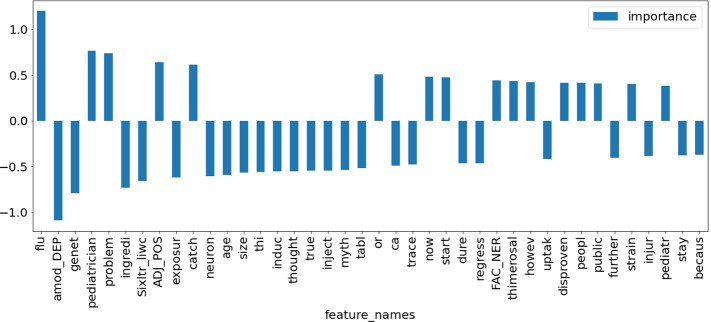
Feature weights retrieved from Logistic Regression model for ’vaccination’ category. Top absolute 40 feature weights are depicted (roughly 30% of all model features)

**Fig. 11:**
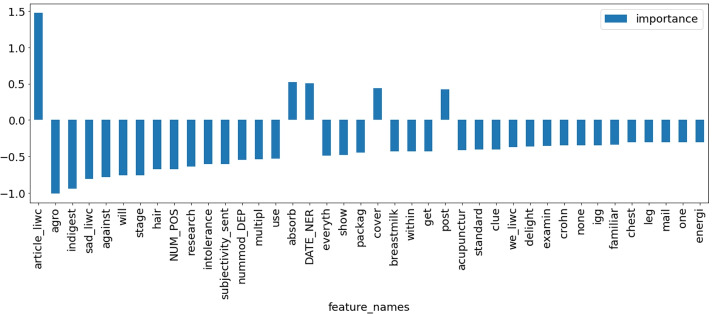
Feature weights retrieved from Logistic Regression model for ’allergy testing’ category. Top absolute 40 feature weights are depicted (roughly 30% of all model features)

**Fig. 12:**
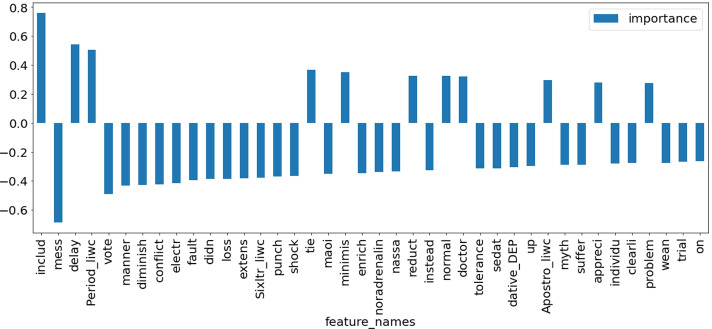
Feature weights retrieved from Logistic Regression model for ’psychiatry’ category. Top absolute 40 feature weights are depicted

**Fig. 13:**
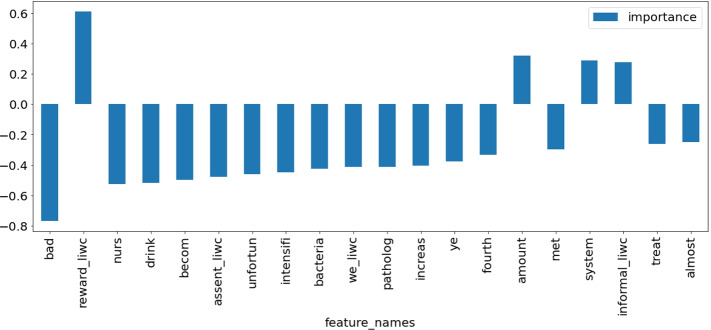
This data is mandatory. Please check.

## Appendix A: filtering classifier models

Table 6 presents models selected by TPOT for each subdomain (category), their performance and the comparison to baseline Logistic Regression.

**Table 6:** Comparison of AUC and weighted F1 performance measures for models selected by TPOT and Logistic Regression for each subdomain

Category	Selected Model (SM)	F1(std) SM	F1(std) LR	AUC(std) SM	AUC(std) LR
A	MLP(50,20) with logistic activation	81(3)	76(5)	87(7)	86(2)
B	MLP(30,20) with ReLU activation	89(2)	80(3)	86(7)	79(6)
C	MLP(50,20) → GradientBoosting → LogisticRegression	77 (3)	82 (3)	69 (5)	81 (5)
D	MLP(50,20) with ReLU activation	78(2)	74(5)	84(4)	83(3)
E	MLP(50,20) + MLP(30,20)	87(3)	84(3)	90(4)	79(6)
F	MLP(50,20) with logistic activation	94(2)	96(2)	85(5)	95(5)
G	GradientBoosting → MLP(50, 20) with ReLU activation	73(3)	81(5)	75(5)	80(9)
H	MLP(50,20) with logistic activation	91(1)	71(17)	85(4)	71(3)
I	MLP(50,20) with logistic activation	88(4)	68(8)	83(2)	67(10)
J	GradientBoosting → MLP(50, 20) with ReLU activation	68(4)	71(3)	71(6)	76(5)

A - heart supplements; B - Antioxidants; C - Cholesterol & statins; D - Vaccination; E - Allergy testing; F - Children antibiotics; G - Diet & Autism; H - Steroids for kids; I - CC vs. Natural Birth; J - Psychiatry

## Appendix B: logistic regression topical models

Below we present feature weights for Logistic Regression models per each topic.

## Footnotes

1 https://github.com/alenabozny/medical_credibility_corpus
